# New Information of the Anatomy and Phytochemical Screening of *Pentaclethra macroloba* (Willd.) Kuntze (Caesalpinioideae-Leguminosae) Seeds

**DOI:** 10.1155/2023/1446972

**Published:** 2023-11-29

**Authors:** Zelina Ataíde Correia, Ely Simone Cajueiro Gurgel, Olivia Ribeiro, Ana Cristina de Andrade Aguiar Dias, Ravendra Kumar, Luis Adriano Santos do Nascimento, Eloisa Helena de Aguiar Andrade, Mozaniel Santana de Oliveira

**Affiliations:** ^1^Graduate Program in Biological Sciences, Concentration Area: Tropical Botany, Federal Rural University of the Amazon and Museu Paraense Emílio Goeldi, Av. Perimetral, 1901, Terra Firme, 66077-830, Coordenação de Botânica, Belém, PA, Brazil; ^2^Institute of Biological Sciences, Federal University of Pará, Belém, PA 66075–110, Brazil; ^3^Department of Chemistry, College of Basic Science and Humanities, G.B. Pant University of Agriculture and Technology, U.S. Nagar, Pantnagar, Uttarakhand, India

## Abstract

*Pentaclethra macroloba* (Willd.), whose common name is “pracaxi,” is naturally found in the Amazon region. The present study is aimed at analyzing the anatomy, seed histochemistry, and chemical composition in fatty acid profile of *P. macroloba* seed oils. Seeds were collected in the cities of Belém, Marituba, and São Domingos do Capim-PA. For the study in light microscopy, scanning electron microscopy, and histochemistry, seeds were sectioned in cross and longitudinal sections of the embryonic axis and fixed in formaldehyde, acetic acid, and 50% ethyl alcohol; neutral-buffered formalin; and formaldehyde and ferrous sulfate and stored in 70% ethyl alcohol. For the anatomical study, the seeds were subjected to the usual techniques of plant anatomy. Histochemical tests were performed on plant material, freehand sectioned, and embedded in histological paraffin with DMSO. The fatty acid profile was determined for gas chromatography (GC-FID). Integument is divided into three strata, monoseriate exotesta, mesotesta formed by several layers of parenchyma cells, and monoseriate endotesta, formed by compressed cells. Cotyledons are composed of thin-walled parenchyma cells with several secretory cavities and secretory idioblasts. The main metabolic classes are lipids, phenolic compounds, carbohydrates, proteins, and alkaloids. The main fatty acids found in *P. macroloba* oil are oleic, behenic, lignoceric, and linoleic. *P. macroloba* seeds have important anatomical characteristics for their circumscription in Leguminosae and also in Caesalpinioideae, and their oil is rich in fatty acids essential to the human diet, providing many benefits to the human health, such as fatty acids belonging to the omega family (linoleic, oleic).

## 1. Introduction

Leguminosae Juss. is a cosmopolitan organism, with about 19,500 species distributed in 770 circumscribed genera in six subfamilies: Duparquetioideae, Cercidoideae, Detarioideae, Dialioideae, Papilionoideae, and Caesalpinioideae [1]. It circumscribes numerous species of economic interest that are used as ornamental, medicinal, food, and green manure plants [2]. Within these, we highlight *Pentaclethra macroloba* (Willd.) Kuntze (Caesalpinioideae), also known as “pracaxi,” “paracaxi,” and “mulateiro” [3].

This species occurs naturally in várzea (floodplain), solid ground, and igapó forests [4–6]. Its geographical distribution includes the Brazilian Amazon, Venezuela, Guianas, and Central American countries, Trinidad and Tobago [7]. Fruits are leguminous, dry, dehiscent, green (immature), dark brown (ripe), woody pericarp, laterally flattened, and falcate; their seeds have a thin, slightly wrinkled integument, green to brown in different maturation stages [8, 9].

From an ecological point of view, it stands out for being a perennial species of constant leaf replacement, a pioneer of fast growth and widely used in the recovery of degraded areas, as it is a N_2_ fixing species, and, consequently, presents a great potential for the restoration of C, N, and F in the soil [10, 11].

Its applicability also includes the use of wood to build furniture, replacing mahogany, andiroba, and freijo [10–12]. The oil from its seeds has great potential due to its medicinal properties, such as healing and anti-inflammatory properties [13, 14], and it is composed of bioactive fatty acids, behenic acids, and oleic acids, which promote moisturizing and emollient action, widely used for skin and capillary treatment [15].

Pracaxi oil also has insecticidal potential against *Aedes (Stegomyia) aegypti* (L.) larvae, a vector of dengue fever, urban yellow fever, chikungunya, and zika virus [16, 17]. Anatomical and histochemical studies make it possible to determine the nature of chemical substances stored in secretory structures, such as idioblasts, isolated, hypertrophied cells larger than neighboring cells, in addition to cavities, secretory channels, and glandular trichomes, as well as to determine reserve compounds and secondary metabolite [18–21].

Several studies have been carried out involving the histochemistry of oilseeds [22–24], as the structural and chemical characteristics of its fruits and seeds constitute safe criteria in taxonomic, phylogenetic, ecophysiological, and biotechnological studies [25, 26].

Currently, a small number of substances present in the *Pentaclethra macroloba* seed oil have been studied, such as oleic, linoleic, and behenic acids, for the treatment of diabetic ulcers, as a healing agent, and in cosmetic products [26, 27]. Therefore, the purpose of this study was to describe the anatomical aspects and to identify the main classes of reserve compounds present in *Pentaclethra macroloba* (Willd.) Kuntze seeds through histochemical tests and the composition of fatty acids.

## 2. Materials and Methods

**Figure 1:**
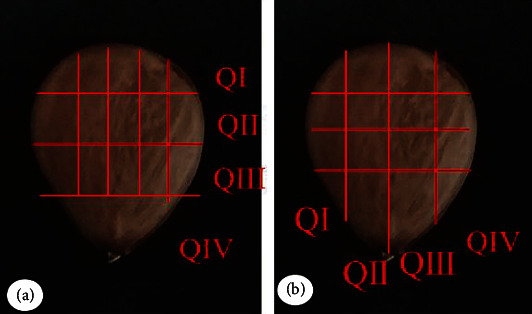
Sections for fixing QI, QII, QIII, QIV, and QV (parts of seeds selected for anatomical sections and histochemical studies): (a) cuts in cross sections and (b) cuts in longitudinal sections.

The *Pentaclethra macroloba* matrices selected for this study are located in the municipalities of Belém, Marituba, and São Domingo do Capim-Pará (Figure 1), located in a várzea forest. The species was identified by Dr. Olívia Domingues Ribeiro, and the ripe fruits were collected in January 2021, from mother plants located in the dense ombrophilous alluvial forest, in the cities of São Domingos do Capim (04/01/2021) (geographic coordinates -1.784919, -47.787479), in Belém (01/11/2021) (-1.470654, -48.44813), and in Marituba (01/14/2021) (-1.461247, -48.293850), all located in the Northeast of the state of Pará. The fertile botanical samples were incorporated into the collections of the Herbarium João Murça Pires (MG), at the Museu Paraense Emílio Goeldi (voucher number MG-243963).

Collections were carried out with the aid of a pruner, before dehiscence, directly in the canopy of the matrices. After collection, the fruits were shade dried, and seed extraction was manually performed, eliminating malformed seeds and seeds with mechanical injuries.

### 2.1. Anatomical Analyses in Light Microscopy (LM) and Scanning Electron Microscopy (SEM)

For anatomical analyses in light microscopy, using a light microscope (Leica® DM750) with a coupled video camera (Leica® ICC50 HD) (Leica Microsystems, Heerbrugg, Switzerland), and scanning electron microscopy, using a scanning electron microscope and LV-FE-SEM TESCAN Mira3 XMU (TESCAN, Brno, Czech Republic) coupled to an EDAX Team (EDAX, Mahwah, USA) and EDS system (EDAX Apollo XPP Silicon Drift Detector), seeds were soaked in distilled water for 24 hours, sectioned transversally and longitudinally to the embryonic axis (Figure 1), fixed in 50% FAA (formaldehyde, acetic acid, ethyl alcohol, 50% 1 : 1: 18 *v*/*v*) for 24 hours under vacuum and stored in 70% ethyl alcohol [28]. The parts of the selected seeds for anatomical and histochemical study are presented in Figures 1(a) and 1(b), where we can observe the cross-sectional and longitudinal cuts, respectively.

For the structural study, seeds previously stored in 70% alcohol were subjected to dehydration in increasing butyl series (tertiary butyl alcohol) for inclusion in histological paraffin with DMSO (Paraplast®, ©Leica Biosystems, USA) [28].

Cross section and longitudinal section of 10 *μ*m to 12 *μ*m were obtained with the aid of a rotating microtome (Leica® RM 2245 model, Leica® Biosystems, Heidelberg, Germany) and then stained with astra blue and safranin [29] and mounted in colorless Canada balsam [30].

For scanning electron microscopy (SEM), cross and longitudinal sections of seeds stored in 70% ethanol were dehydrated in a growing ethylic series [31] and processed in a critical point dryer using CO_2_ as transition liquid, fixed with graphite on metal supports (studs), and metallized with carbon and gold [32].

### 2.2. Histochemical Analysis

Seeds were fixed for compound detection: (i) hydrophilic (FAA50 for 24 hours [28], (ii) lipophilic (NBF—neutral-buffered formalin for 48 hours) [33], and (iii) total phenolics (SFF—formaldehyde, ferrous sulfate, and distilled water for 48 hours) [28] and later stored in 70% ethyl alcohol.

Histochemical tests were applied to the exotesta cuticle and to the secretory structures present in the integument, cotyledon mesophyll, and embryonic axis, performed on freehand sectioned samples, and embedded in histological paraffin with DMSO [34].  Table 1 shows the tests performed to detect the main groups of metabolites.

Control tests were performed according to the recommendations of the respective authors of the applied tests. For each test performed, samples were assembled and photographed before the reagent (blank) was applied, aiming to identify the seed natural appearance.

Photomicrographs in light microscopy and histochemistry were obtained using a microscope (Leica DM6B, Germany W) with a coupled digital camera (Leica Application Suite, LAS, V4. 12) duly calibrated with micrometer slides according to the manufacturer, located in the Microscopy Laboratory at the Botany Coordination of the Museu Paraense Emilio Goeldi Research Campus. And the electromicrography was obtained with a TESCAN Mira3 microscope, with micrometric scales projected under the same optical conditions, located in the Scanning Electron Microscopy Laboratory of the Museu Paraense Emílio Goeldi.

### 2.3. Phytochemical Analysis

Fixed oil extraction was carried out by using a Soxhlet-type apparatus, with 10 g of seeds and 150 mL of hexane as solvent [44]. Extraction was performed for 5 hours. Subsequently, lipid esterification was performed according to [45].

Esterification was performed using the AOCS Official Method Ce 2-66 [46], to destroy epoxy, hydroperoxy, cyclopropenyl, cyclopropyl, and possibly hydroxyl and acetylenic fatty acids for the preparation of fatty acid methyl esters [47]. The oils were analyzed for chemical composition using gas chromatography coupled to a flame ionization detector (GC-FID), as described by [48]. Fatty acid composition was determined using a gas chromatograph (Varian model CP 3380) equipped with a flame ionization detector and a CP-Sil 88 capillary column (60 m in length, 0.25 mm internal diameter, and 0.25 *μ*m film thickness; Varian Inc., USA). This method considers the conversion of fatty acids into fatty acid methyl esters (FAME).

The gas chromatograph used FID detector and injector (split ratio 1 : 100), temperatures of 250°C, and helium as carrier gas at a 0.9 mL/min flow rate. Column temperature was adjusted to 80°C for 4 min and increased to 205°C at a 4°C/min rate. Varian Star 3.4.1 software was used for standard fatty acid chromatograms and known mixtures (Nu-Chek-Prep, Inc., USA) to quantify fatty acids. Fatty acid content was expressed as relative percentages of total fatty acids. In general, three injections (*n* = 3) were performed, and the samples did not show standard deviation.

## 3. Results

### 3.1. Seed Anatomical Description

**Figure 2:**
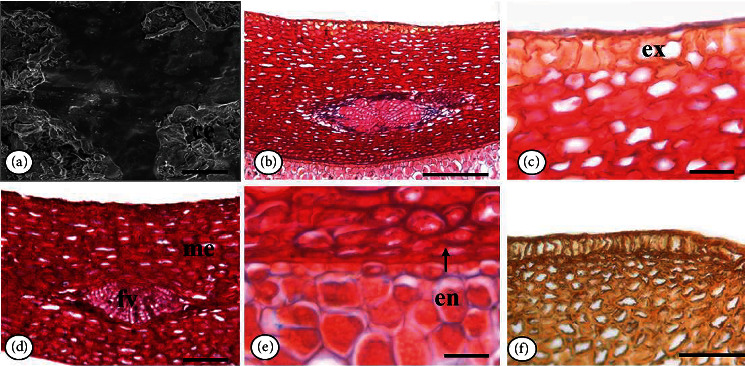
*Pentaclethra macroloba* (Willd.) Kuntze seed integument. Cross section: external surface in scanning electron microscopy (SEM) (a); general view of the integument in light microscopy (LM) (b); exotesta detail (LM) (c); mesotesta detail (LM) (d); endotesta detail (LM) (e); integument in longitudinal section (LM) (f). Caption: ce: wax; en: endotesta; ex: exotesta; me: mesotesta; fv: vascular bundle. Bar: 20 *μ*m (a); 50 *μ*m (c, f); 100 *μ*m (b, d, g).

In scanning electronic microscopy, we observed wax plaques on the integument surface (Figure 2(a)).

The integument is composed of exotesta (epidermis), mesotesta (mesophyll), and endotesta (internal epidermis) (Figure 2(b)). In the cross section, exotesta is monostratified with juxtaposed cuboidal cells, with different sizes and thick walls covered by a smooth and thin cuticle. In longitudinal section, exotesta cells have the same shape as in cross section (Figure 2(c)).

Mesotesta is formed by approximately 18-20 layers of parenchyma cells that are large and horizontally elongated, with thick walls, which decrease in size in the inner layers (Figures 2(d) and 2(e)). In the central region of the mesotesta, there are amphicribal vascular bundles that can be observed in different regions of the integument (Figure 2(d)). In cross section, mesotesta cells have a slightly rounded shape (Figure 2(f)).

Endotesta consists of a monoseriate layer of rectangular, compressed cells with thickened walls (Figure 2(e)).

**Figure 3:**
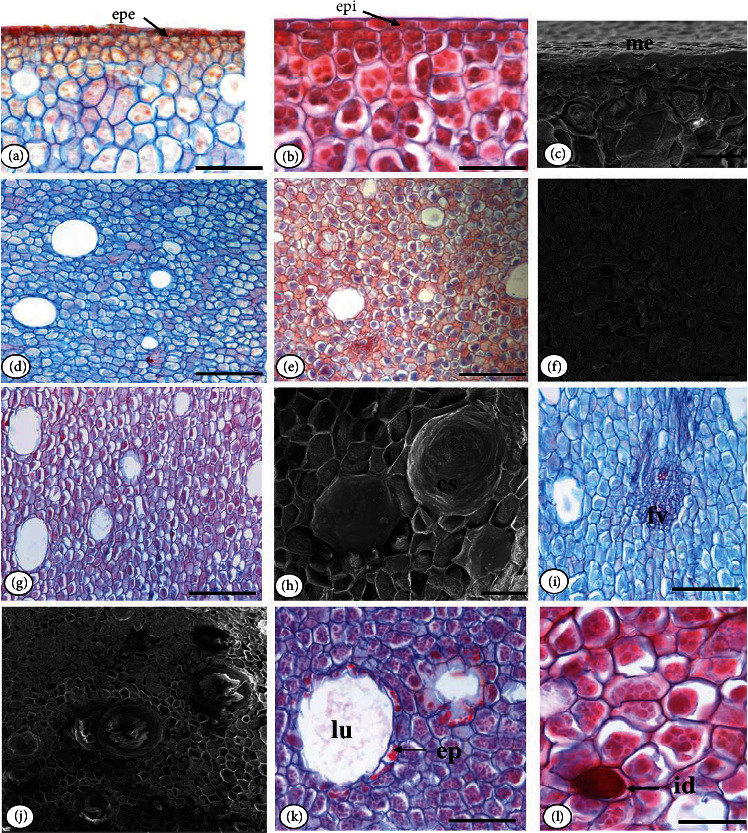
*Pentaclethra macroloba* (Willd.) Kuntze seed cotyledons: (a) detail of the cotyledon outer epidermis in cross section (LM); (b) detail of the cotyledon inner epidermis in cross section (LM); (c) detail of the mesoderm in longitudinal section (SEM); (d) cotyledon mesophyll in cross section (LM); (e) idioblasts in the cotyledon mesophyll (LM); (f) cotyledon cells (SEM); (g) cotyledon mesophyll in longitudinal section (LM); (h) secretory cavities in longitudinal section (LM); (i) detail of the vascular bundle; (j) secretory cavities in cross section (SEM); (k) detail of the cavity in light microscopy (LM); (l) secretory idioblast. Caption: cs: secretory cavity; epe: outer epidermis; epi: internal epidermis; ep: secretory epithelium; id: idioblast; lu: lumen; me: mesoderm. Bar: 20 *μ*m (c, f, h, j); 100 *μ*m (a, b, d, e, g, i, k, l, d). SEM: scanning electron microscopy; LM: light microscopy.

In cross section, the cotyledon epidermis is monoseriate, with rectangular cells, with thickened walls, and covered by a smooth cuticle (Figures 3(a) and 3(b)). In longitudinal section, the monostratified mesoderm with an external periclinal wall covered by wax can be seen (Figure 3(c)).

The cotyledon mesophyll is filled with parenchyma tissue with reserve characteristics, being consisting of polygonal, heterodimensional cells with thin walls (Figures 3(d)–3(f)). In longitudinal section, mesophyll cells present a slightly elongated format in relation to that observed in cross sections (Figures 3(g) and 3(h)).

The vascular tissues are organized in collateral vascular bundles, dispersed in the cotyledon mesophyll (Figure 3(i)). In cross section, the secretory cavities are composed of monoseriate secretory epithelium, formed by thin-walled tabular cells, delimiting the isodiametric lumen (Figures 3(j) and 3(k)). Secretory idioblasts are found throughout the mesophyll (Figure 3(l)).

**Figure 4:**
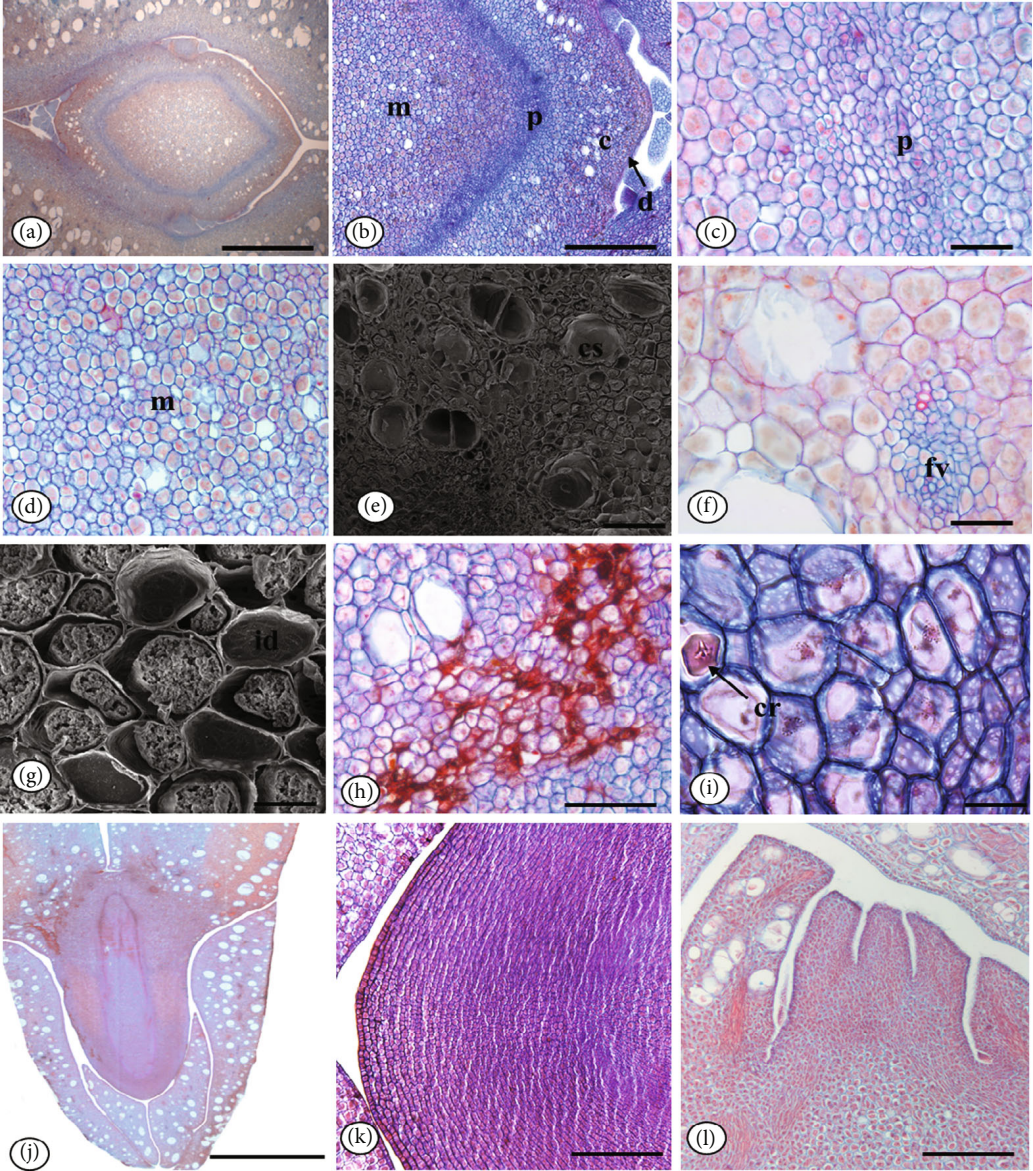
*Pentaclethra macroloba* (Willd.) Kuntze seed embryonic axis: (a) general view of the axis in cross section (LM); (b) detail of the embryo in cross section (LM); (c) detail of the procambium (LM); (d) detail of the medullary region; (e) secretory cavities (LM); (f) vascular bundle (LM); (g) SEM; (h) detail of cavities without cross section (LM); (i) prismatic crystal (LM); (j) general view of the embryo in longitudinal section (LM); (k) hypocotyl radicle in longitudinal section (LM); (l) plumule in longitudinal section (LM). Caption: c: fundamental cortical meristem; cs: secretory cavity; d: protoderm; fv: vascular bundle; m: medullary ground meristem; p: procambium; cr: crystals. Bar: 50 *μ*m (i), 20 *μ*m (e, g), 100 *μ*m (a, b, c, d, f, h, k, l), and 500 *μ*m (j). SEM: scanning electron microscopy; LM: light microscopy.

The embryo is cotyledonary, and in cross section, the protoderm of the embryo is monostratified with rectangular cells and thin periclinal walls (Figures 4(a) and 4(b)).

The cortical ground meristem comprises approximately 20 layers of thin-walled, slightly elongated, heterodimensional polygonal cells, where an abundance of secretory cavities and idioblasts can be seen (Figure 4(b)). The procambium shows little cell differentiation, consisting of approximately 12-15 slightly elongated polygonal cells smaller than cortical and medullary tissue cells (Figures 4(b), 4(c), and 4(e)).

The medullary fundamental meristem occupies the largest volume of the embryo, formed by heterodimensional polygonal cells, with thin walls, and throughout the length of the medulla, there are secretory idioblasts (Figure 4(d)). Collateral vascular bundles are located in the region of the embryo cotyledons (Figure 4(f)).

In the embryonic axis, secretory idioblasts were identified (Figure 4(g)); the secretory cavities are constituted by a monoseriate secretory epithelium, formed by tabular cells with thin walls, isodiametric lumen similar to those found in the cotyledon mesophyll (Figure 4(h)). In the cortical and medullary tissue regions, small prismatic crystals are observed (Figure 4(i)).

The embryo in longitudinal section consists of two cotyledons and the elliptical-basal axis, straight, with epicotyl, hypocotyl, and radicle (Figure 4(j)).

The root cap presents monostratified epidermis formed by cuboidal cells and covered with smooth and thin cuticle (Figure 4(k)). In longitudinal section, the plumule is poorly differentiated (Figure 4(l)).

### 3.2. Seed Histochemistry

**Figure 5:**
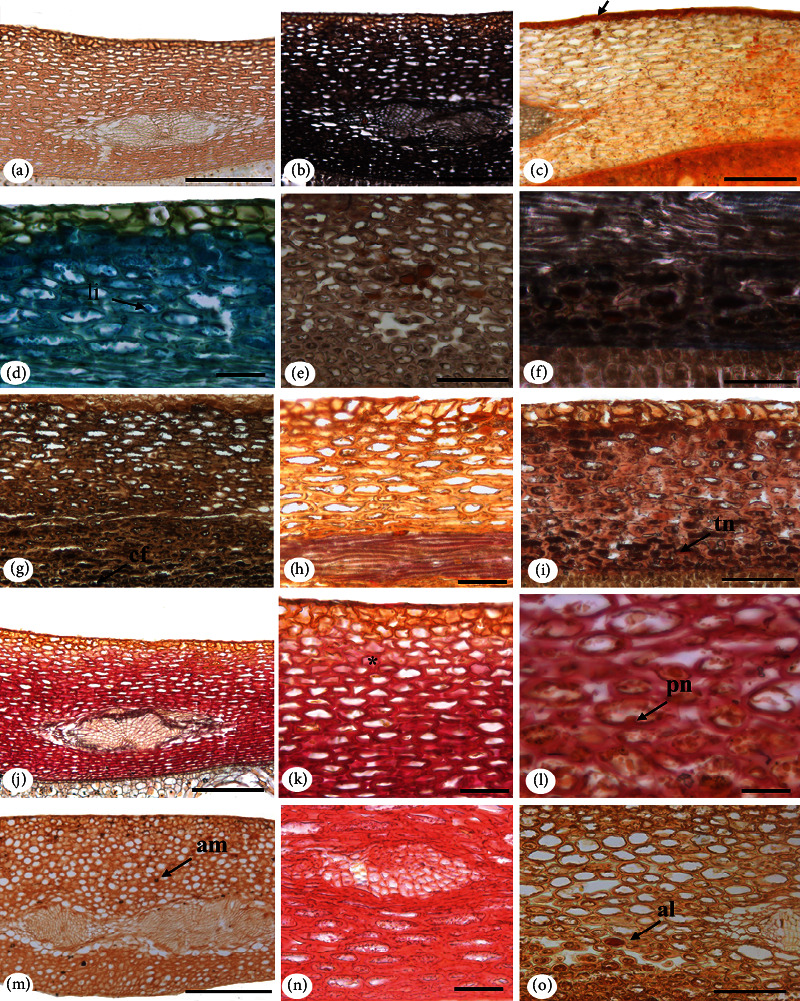
Histochemical tests, positive reactions for different classes of metabolites present in the *Pentaclethra macroloba* (Willd.) Kuntze seed integument: (a) integument not submitted to tests (LM); (b) detection of total lipids with Sudan black (LM); (c) detection of total lipids with Sudan III (arrow) positive reaction for lipids in the cuticle (LM); (d) positive reaction for acid lipids (LM); (e) positive reaction for terpene oil resin in idioblasts (LM); (f) terpene reaction in idioblasts (LM); (g) positive reaction for total phenolic compounds; (h) positive reaction for lignin in the vascular bundle (LM); (i) positive reaction for tannin (LM); (j) positive reaction for pectin (LM); (k) positive reaction for pectin in the cuticle of the exotesta (arrowhead) and acidic mucilage (^∗^) (LM); (l) positive reaction for neutral polysaccharides (LM); (m) starches (LM); (n) total proteins (LM); (o) idioblast with alkaloids (LM). Caption: al: alkaloids; am: starch; cf: phenolic compounds; li: lipids; tn: tannin; pn: total polysaccharides. Bar: 50 *μ*m (d, l, n, h), 100 *μ*m (b, c, e, f, g, h, i, o), 100 *μ*m (a), and 200 *μ*m (m).

**Figure 6:**
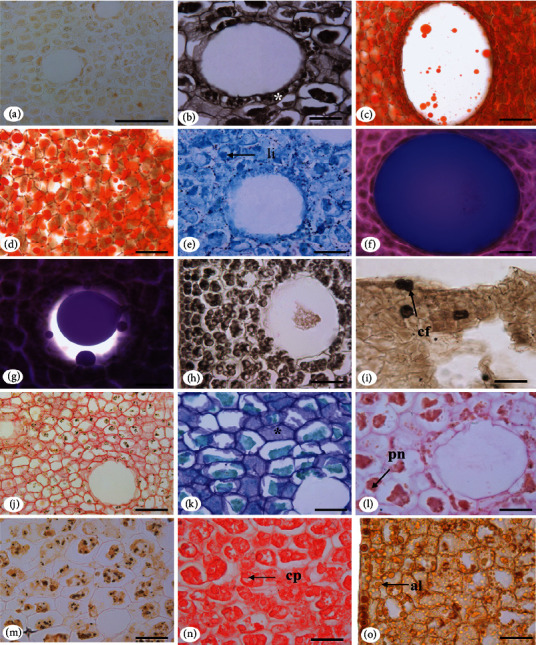
Histochemical tests applied to *Pentaclethra macroloba* (Willd) Kunze seed cotyledons: (a) cotyledon mesophyll in white (LM); (b) positive reaction for total lipids with Sudan black evidenced, droplets in the secretory epithelium of the cavity (^∗^) (LM); (c) positive reaction for total lipids with Sudan III (LM); (d) total lipids in the cotyledon mesophyll (LM); (e) positive reaction for acid lipids (LM); (f) positive reaction for terpenes, showing essential oil (LM); (g) cavity secreting terpenes (LM); (h) phenolic compounds in the lumen and in the secretory epithelium of the cavity, detected by the SFF fixative (LM); (i) phenolic idioblasts detected by ferric chloride (LM); (j) pectic compounds in cell walls (LM); (k) idioblasts with acidic mucilage (^∗^) (LM); (l) neutral polysaccharides (LM); (m) starch (LM); (n) total proteins; (o) alkaloids (LM). Caption: al: alkaloids; cf: phenolic compounds; cp: protein bodies; li: lipids; pn: neutral polysaccharides. Bar: 50 *μ*m (b, c, d, e, f, g, h, i, j, k, l, m, n, o); 100 *μ*m (a).

**Figure 7:**
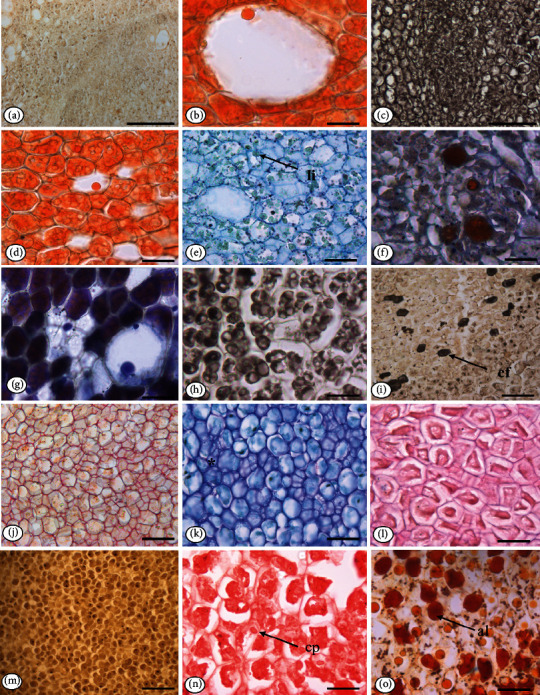
Histochemical tests applied to the embryonic axis of *Pentaclethra macroloba* (Willd): (a) cross section showing the blank embryo (LM); (b) positive reaction for total lipids with Sudan III (LM); (c) positive reaction for total lipids with Sudan black (LM); (d) total lipids in the embryo cotyledon (LM); (e) positive reaction for acid lipids in the embryo cotyledons (LM); (f) positive reaction for terpenes, showing oil resin in the idioblasts in the procambium region (LM); (g) terpenes in the lumen of the secretory cavity, showing essential oil (LM); (h) phenolic compounds detected by the SFF fixative (LM); (i) phenolic idioblasts detected in the cortical ground meristem (LM); (j) pectic compounds in the cell walls (LM); (k) idioblasts with acidic mucilage in the medullary region (^∗^) (LM); (l) neutral polysaccharides; (m) starch (LM); (n) total proteins (LM); (o) alkaloids (LM). Caption: al: alkaloids; cf: phenolic compounds; cp: protein bodies; li: lipids. Bar: 50 *μ*m (b, c, e, f, g, h, i, k, l, n, o); 100 *μ*m (a, d, j, m).

Positive reactions were observed for lipids, terpenes, total phenolic compounds, tannin, mucilage, pectin, polysaccharides, starch, proteins, alkaloids, lignin, and calcium oxalate crystals in the embryonic axis region. These results are organized in Table 2 and their reactions in Figures 5–7.

### 3.3. Analysis of Fatty Acids

Results referring to the fatty acid composition of the three studied populations demonstrated that *P. macroloba* oil is rich in unsaturated and saturated fatty acids, especially the oleic unsaturated fatty acid (59%), while behenic acid with concentration ranging between 13% and 14% among the analyzed populations was the main saturated fatty acid. Lignoceric and linoleic fatty acids showed concentrations ranging from 9% to 11% and 7% to 9%, respectively. To a lesser extent, stearic acid (2.5% to 3%), arachidic acid (0.76 to 3%), palmitic acid (0.94 to 2.3%), erucic acid (0.80% to 0.94%), and tricosanoic acid (0.12%) were found in all populations.

Some differences in the composition of fatty acids were observed, such as the presence of palmitoleic acid present in the chemical composition of the Belém population and Marituba population and absent in the chemical composition of São Domingos do Capim population. Margaric acid was only present in the population of Marituba, and linolenic acid was absent in the population of Belém (Table 3).

## 4. Discussions

In the current investigation, we examined the structural characteristics evident in the integument of *P. macroloba*. However, the exotesta cell invagination process, which results in the formation of cavities reported by [3], was not noticed in the present study.

Anatomically, *P. macroloba* seeds do not have the complex anatomical structure normally described for the Leguminosae family, such as an exotesta formed by palisade cells, called Malpighian cells (macrosclereids), light line, mesotesta formed by hourglass cells (osteosclereids), and endotesta by several layers of parenchyma cells [49–53].

The anatomical characteristics of the *P. macroloba* integument were also observed for some seeds of species belonging to the genus Bauhinia L. by [54], in which they observed that the species *Bauhinia aculeata* L., *Bauhinia purpura* L., and *Bauhinia variegata* L. have an exotesta formed by cuboidal cells and a mesotesta by parenchyma cells, while other species of the genus follow the pattern already described for the family.

The structural characteristics observed in the *P. macroloba* integument may suggest the absence of physical dormancy for the species, since Orwa et al. (2009) describe the *P. macroloba* seeds as intermediate, unlike what occurs in some species of Bauhinia L. [54, 55], *Piptadenia moniliformis* [56], and *Cenostigma* Tul. [57]. Thus, the anatomical organization of the *P. macroloba* integument can provide important subsidies for the species identification in the Leguminosae family, as observed by [58] when anatomically comparing seed integuments of *Senna occidentalis* (L.) Link (Fabaceae-Caesalpinioideae) and *Phyllanthus niruri* L. (Euphorbiaceae).

The vascular bundles present in the *P. macroloba* mesotesta can be found throughout the entire length of the integument, quite different from what was described for *Senna spectabilis* (D.C.) Irwin & Barneby var. excelsa (Schrad) Irwin & Barneby, in which they remain in the micropyle and chalaza [59]. As for the embryo, it consists of two cotyledons and the elliptical-basal axis, straight with epicotyl and short radicle hypocotyl; some of these characteristics were also observed by [60] when studying species belonging to the Leguminosae subfamilies. These features are commonly found in the Caesalpinioideae subfamily [49].

Histochemical analyses of *P. macroloba* seeds revealed the presence of lipids and pectic compounds in the cuticles covering the exotesta and in the epidermis of the seed cotyledons. The cuticle that covers all plant epidermis cells can act as a protective barrier against excessive water loss and entry of pathogens, insects, and agrochemicals [61, 62].

The presence of cuticle in the seeds of species belonging to the Leguminosae is constantly described in the literature [63–65]. The cuticles present in plants are made up of lipophilic cutin and hydrophobic compounds called waxes, in addition to a varied number of polysaccharides [66–68]. It is worth mentioning that both the presence and the ornamentation of the cuticle have a great taxonomic value for the species [69].

Lipid compounds are present in all regions of *P. macroloba* seeds, in the cotyledon mesophyll and in the embryonic axis, and are produced and stored in the secretory cavities, as well as in idioblasts. This information regarding the lipid compound production and localization for the species under study was also pointed out by [8].

In oilseeds, it is common to store lipids and fats in lipid bodies usually found in reserve tissues, such as cotyledons or endosperm, providing carbon during cellular respiration and acting as an energy source in the germination process, in addition to contributing to the seedling adaptation to less illuminated environments [70, 71].

Acidic lipids are found in abundance in *P. macroloba*; the acidic nature of lipids is directly linked to the fatty acids present in the oil; the reserve lipids found in seeds are triglycerides, and the most common fatty acids are oleic acids, linoleic acids, and linolenic acids [72, 73]. There are differences regarding the lipid composition of *P. macroloba* seeds; the positive reaction to the Nadi reagent showed the presence of terpenes of different molecular weights, such as essential oils and resin oil.

Essential oils are compounds (mono- and sesquiterpenes and phenylpropanoids) that attribute organoleptic characteristics and have phytotherapeutic actions in the human body—by presenting analgesic, antibacterial, and antioxidant action [74–76]—while resins are (di-, tri-, or tetraterpenes) and provide the plant with protection against herbivory [77].

In *P. macroloba*, phenolic compounds are present in all regions of the seed. In the cotyledon mesophyll and in the embryonic axis, they can be observed in the idioblasts and in the secretory cavities. The presence of phenolic compounds in the cavities proves the mixed nature of the chemical substances that the oil cavities produce, in general, terpenes and phenolic compounds. The same secretory structure can secrete different chemical substances depending on its origin or developmental stage of the tissue where it is located [78, 79].

Phenolic compounds can be found in several plant species [80]. This chemical group also includes flavonoids and tannins. Histochemical tests for flavonoids were not performed in this study, as tannin is only present in the *P. macroloba* seed integument. Tannins have several benefits, such as antioxidant and antimicrobial benefits [81]. Flavonoids are antioxidant, anti-inflammatory, antimicrobial, and antiallergic [82].

In plants, phenolic compounds represent the main bioactives present in oils, and they act to protect against microorganisms, especially fungi and herbivores [83–85], in addition to participating in the control and absorption of water and in the entry of oxygen in some seeds [86, 87]; besides, integument coloration has also been linked to phenolic compounds [88].

Starch is distributed throughout all regions of the *P. macroloba* seed, similarly to what has been observed for other species of Leguminosae [88, 89]. The starch stored in the seeds is an insoluble polysaccharide consisting of amylose and amylopectin [90]. In the *P. macroloba* seeds, a large accumulation of lipids was observed; however, for some Leguminosae seeds, the accumulation of lipid compounds is inversely proportional to the starch [89].

Therefore, reserve carbohydrates stored in some legume seeds can be applied as taxonomic characteristics, since they do not occur regularly between subfamilies [88, 91].

The proteins present in *P. macroloba* seeds are lipoproteic, as they divide the cell cytoplasm with lipids. The presence of proteins in the cotyledons for some Leguminosae species may be related to desiccation tolerance [89, 92]. Proteins are the main source of nitrogen and sulfur and act in the synthesis of other compounds [93]. Alkaloids, in turn, can be found in all regions of *P. macroloba* seeds. Alkaloids play an important role in protecting against herbivory, as well as having medicinal properties, such as anesthetic, antitumor, myorelaxant, and antimicrobial action [94, 95].

Regarding the composition of fatty acids, we verified that *P. macroloba* oil is rich in saturated and unsaturated fatty acids. A composition similar to that described in this study was reported by [14, 96]. However, lower concentrations were described for oleic acid (47%) and higher concentrations for behenic acid (22%) and arachidic acid (12%) by [97]. Teixeira et al. [98] also reported a lower value of oleic acid (53%). Furthermore, Pereira et al. [99] reported an increase for linolenic acid (37%).

Variations in the chemical concentrations of the oils may be related to the origin of the seeds, as well as natural factors and methods of extracting the seeds for oil production [100]. In the present study, lauric and myristic acids were not found in the composition of fatty acids, already reported in previous studies [14, 96, 97, 99]. As well as the presence of margaric fatty acid in the composition of the oil from the *P. macroloba* population from the Municipality of Marituba, it had not yet been reported in other studies. Regarding erucic acid, its concentration ranges from 0.80% to 0.94% among the studied populations of *P. macroloba*, a value close to that reported by [99].

## 5. Conclusion

The study of *Pentaclethra macroloba* seeds has revealed significant anatomical characteristics that are crucial for distinguishing this species within the Caesalpinioideae family. Notably, the unique structure of the integument sets it apart from the typical pattern observed in legume species. Furthermore, the investigation into anatomical, histochemical, and phytochemical features has contributed to expanding our knowledge of this species. These findings are particularly valuable for supporting research in the field of seed technology, particularly in areas such as storage and biotechnology. One noteworthy aspect of the research is the recognition of the substantial oil content in *Pentaclethra macroloba* seeds. This oil serves as a rich source of bioactive compounds that have been shown to have health benefits. These findings have the potential to drive further research into the utilization of these bioactives for various applications, including medicinal and dietary purposes. Additionally, this study underscores the importance of species conservation and the sustainable use of *Pentaclethra macroloba*. By gaining a deeper understanding of the species' characteristics and potential applications, we can better appreciate the ecological and economic significance of preserving and responsibly utilizing this plant. Overall, the findings shed light on the value of biodiversity and the potential benefits it can offer to both science and society.

## Figures and Tables

**Table 1:** Histochemical tests applied to detect the main classes of metabolites.

Metabolic groups	Reagents	Reaction
Alkaloids	Dragendorff's reagent [35]	Reddish brown
Starch	Lugol's solution [28]	Black
Total phenolic compounds	Ferric chloride III SFF [28]	Black 1932
Calcium oxalate crystals	Hydrochloric acid [36]	Total dissociation of crystals
Lignin	Acid phloroglucinol [28]	Pink
Acid lipids	Nile blue A [37]	Blue
Total lipids	Sudan III [28]	Orange
	Sudan black [38]	Black
Acidic mucilage	Toluidine blue pH 4.7 [39]	Purple
	Ruthenium red [28]	Pink
Pectic compounds	Ruthenium red [28]	Pink
Total polysaccharides	Schiff test/periodic acid-PAS [40]	Pink
Total proteins	Xylidine ponceau [41]	Red
Tannin	Vanillin-hydrochloric acid [42]	Red
Terpene	Nadi reagent [43]	Blue (essential oil)
		Red (resin oil)
		Purple (mixture)

**Table 2:** Results of histochemical tests performed on *Pentaclethra macroloba* seeds.

Reagent	Seed structure					
	Metabolites	Integument	Cotyledon mesophyll	Embryonic axis	Secretory cavities	Idioblasts
Dragendorff	Alkaloids	+	+	+	-	+
Lugol	Starch	+	+	+	-	-
Iron chloride	Phenolics compounds	+	+	+	+	+
Ruthenium red	Pectin	+	+	+	-	-
Hydrochloric acid	Calcium oxalate crystals	n	n	+	n	+
Acid phloroglucinol	Lignins	+	n	n	n	n
Nile blue A	Acid lipids	+	+	+	-	+
Sudan III Sudan black	Total lipids	+	+	+	+	+
Toluidine blue	Acidic mucilage	+	+	+	-	+
PAS	Neutral polysaccharides	+	+	+	-	-
Xylidine ponceau	Total proteins	+	+	+	-	-
Vanillin-hydrochloric acid	Tannin	+	-	-	-	+
Nadi	Terpenes	+	+	+	+	+

Note: n: not applied; +: positive reaction; -; negative reaction.

**Table 3:** Chemical composition and fatty acid content of fixed oil extracted from *P. macroloba* seeds.

	Compounds	São Domingos	Marituba	Belém	Ret. time
1	Palmitic	2.3103	2.1946	1.7464	17.589
2	Palmitoleic	—	0.0734	0.0856	17.843
3	Margaric	—	0.0405	—	18.541
4	Stearic	3.1020	2.5301	2.5948	19.528
5	Oleic	59.2053	59.4308	58.6091	19.755
6	Linoleic	7.9605	9.1528	8.4813	20.098
7	Linolenic acid	0.3205	0.0643	—	20.632
8	Arachidic	2.5002	0.7677	1.4979	21.402
9	Behenic	13.6552	13.5324	14.7712	23.222
10	Erucic	0.8492	0.9401	0.8051	23.439
11	Tricosanoic	0.1273	0.1190	0.1363	24.354
12	Lignoceric	9.9696	11.1544	11.2723	25.896

## Data Availability

Data are available on request from the corresponding author.
